# Observations on the Epidemic Yellow Fever of Natchez, and of the South-west

**Published:** 1842-05

**Authors:** John W. Monette

**Affiliations:** Washington, Mississippi


### WESTERN JOURNAL

OF

### MEDICINE AND SURGERY.

---

MAY, 1842.

---

ART. I.—*Observations on the epidemic Yellow Fever of Natchez, and of the South-west.* By JOHN W. MONETTE, M. D., of Washington, Mississippi.

(*Continued.*)

EPIDEMIC YELLOW FEVER AS IT PREVAILED IN THE SOUTH-WEST IN THE SUMMER AND AUTUMN OF 1839.

As the summer and autumn of 1839 must constitute a memorable epoch in the history of this pestilence, not only in the West Indies but also in the southern portion of the United States, we propose to give a general sketch of the season, as well as of some other circumstances which have contributed much towards its unusual prevalence in this portion of our country. We believe that yellow fever never has prevailed epidemically, at the same time, in so many seaports and inland trading towns of the United States, as it did in the summer of 1839. Scarcely a southern port or trading town, having direct commercial intercourse with the infected ports of the West Indies, or Mexico, escaped a visitation of epidemic yellow fever, more or less severe; and almost every inland town, having direct and unrestricted commercial intercourse with the *ports*, after they became infected, became successively infected also.

The first appearance of this disease in the United States, during this summer, was invariably in the maritime or commercial ports; and the first cases were invariably among the shipping in port, and especially among those which were direct from infected West India or Mexican ports. In every instance the disease for several weeks was confined exclusively to the shipping, before it began to spread among the resident population. This fact is abundantly established by the concurrent statements of the public press in all the infected ports. In no port of the United States was a single case of yellow fever seen, even on board the vessels, until after it had been prevailing with great mortality for several weeks in the West India and Mexican ports.

The yellow fever began to rage in the port of Havana and Vera Cruz, early in the month of May: other ports became likewise infected nearly about the same time, such as Matanzas, St. Jago and others. The disease in all these ports had become epidemic before the 1st of June. In Havana it had become epidemic on the 24th of May, and on the 28th of May several American captains and seamen had died of it in the port of Havana.* During the month of June it raged with unusual fatality, and the interments at that port for that month were 488.

It was late in August when the yellow fever began to decline; the Havana papers of August 10th state, that "the yellow fever is still very bad among the shipping." Ten days later, they state that "the sickness among the strangers has almost subsided for want of subjects."† "At Vera Cruz, it continued to rage with unabated violence," until the 16th day of October, "when there were more than 400 cases in the hospital."‡

*N. O. Bulletin. †Idem, Aug. 10th and following. ‡Idem for Sept. & Oct.

In Charleston S. C. yellow fever cases were known to be among the shipping from the West Indies early in June. On the 7th of June, Dr. Strobel, the port-physician, reported three cases of yellow fever on board the brig Burmah, five days from Havana. Two of them died the next day.* On the 10th, other cases were reported on board other vessels; on the 12th the Briganza arrived from Havana with several yellow fever cases on board. Other vessels continued to arrive at Charleston with other cases on board until the first week in July, when it began to spread rapidly among the vessels and produced much alarm. During the whole of this time the resident population was perfectly healthy, until about the 10th of July, when the disease began to spread among the people near the wharves; and in ten days afterwards it began to prevail over other parts of the city.

About this time a vessel from Havana with yellow fever on board arrived at the quarantine ground in New York. The vessel was not permitted to enter the port; and no disease was communicated to the residents of the city. A few days afterwards an infected vessel from Havana, arrived in Portland, Maine, after losing several of her crew on the voyage. Several deaths by yellow fever soon after occurred in that place in persons who had been on board this vessel while at the Portland wharf.†

In *New Orleans* vessels from Havana arrived almost daily during the season. About the last of June, several cases of yellow fever had occurred among the shipping from the West Indies. Towards the close of July cases of yellow fever among the shipping were more frequent, and by the 1st of August about 25 cases had been received into the Charity Hospital. The cases among the shipping increased rapidly in the first ten days of August, during which time the disease was gradually insinuating itself among the resident population, contiguous to the wharves and shipping. On the 12th it

---

*See his report—Charleston Mercury. †See Eastern Argus, Portland—also the Bulletin for July 26th, 1839.

was admitted to be epidemic in the city. From the first of August up to this time the number of admissions into the hospital varied from 8 to 15 daily.

Mobile, Savannah, and other ports trading with the West Indies, were successively infected in the order of their commercial importance; while not a single *inland* town was infected, or was known to contain a case of yellow fever, from New York to Louisiana. The whole interior country, including towns and villages, was remarkably healthy, and exempt from all the ordinary bilious diseases of the season. In no inland town did yellow fever make its appearance either in sporadic cases, or as an epidemic, until at least ten days after it had been epidemic in the nearest commercial port; and the order of its appearance in the towns on the Mississippi, was exactly in proportion to the amount of direct communication by steamboats, with New Orleans, after yellow fever was epidemic in that city. The same was true of the small towns around the northern shore of the Gulf of Mexico, from St. Joseph's and Tampa Bay to the Teche, and even to Galveston in Texas. The town of Augusta, in Georgia, in like manner became infected after the yellow fever became epidemic in Charleston and Savannah.

As we progress, we propose to trace the connection between the disease in the maritime ports and the West India ports; as well as between the disease in the former, and the interior towns dependent on them for commercial supplies.

Although we do not propose to throw much light upon the treatment of this intractable disease—this West India pestilence, which, in its worst form, derides the skill of the whole medical faculty, and all the discoveries of science; yet we propose to do what is far better. We propose to point out prudential measures and precautions, which will more effectually protect our commercial ports from the ravages of this disease, than all the improvements which have been introduced into the healing art for ages past;—the one to excel the other, as far as prevention excels cure.

Independently of any extrinsic cause, such as *imported in-*

*fection*, there was, during the summer of 1839, "a general atmospheric predisposition," operating over a wide extent of country, and which prepared the local atmosphere of towns and cities for the easy dissemination of yellow fever as an epidemic. This atmospheric predisposition is nothing more nor less than the "epidemic constitution" of Professor Caldwell of the Louisville Institute. This condition of the atmosphere does exist, as he very properly contends. The peculiarity of this constitution we shall endeavor to illustrate. It is that condition of the air, which appears to be peculiarly adapted to the production of yellow fever infection—a condition which exists sometimes from June to August in the middle and northern States, and from July to October in the southern states. It exists, in all probability, only at the times and places above designated; because there is no instance on record where yellow fever *assumed* its epidemic character in the United States, north of lat 40°, after August or before June; or in which it first assumed that character, south of 35°, before July or after the month of October.

This peculiarity, as we have already shown at large, consists, as we suppose, chiefly in a continued, hot, calm, and sultry condition of the general atmosphere; at which time, the air of cities and towns, being more confined than the common country air, and being respired by thousands of persons constantly for many days together, becomes thoroughly charged or contaminated by the effluvia thrown off by a population of healthy human beings. This condition of the local atmosphere adapts it to the dissemination of yellow-fever infection; and when a sufficient quantity of infected air from an infected town or city, is introduced into this local atmosphere, it assimilates the whole of the contaminated atmosphere, and thus infects a portion of a town or city. Several patients laboring under yellow fever, confined in a circumscribed portion of this air, at a high summer temperature, for several days, will impart to that portion of the local atmosphere certain properties, which will cause it, after being secluded from ventilation for a few days longer, to assume the properties of the most virulent yellow-fever infection.

Upon these principles we think it demonstrable that yellow fever has been repeatedly, and may be again introduced from infected towns, into those which were previously perfectly healthy; and, consequently, that suitable precautions at the proper time may and will prevent a recurrence of such epidemic.

The state of the *weather* during the whole summer of 1839 was *extraordinary*, in all the southern portion of the United States. Even the month of April was uncommonly hot and dry. The same condition of the atmosphere continued to exist during the succeeding months of May, June, and July, with a few light and transient showers, the latter only south of lat. 31° in the lower valley of the Mississippi. From 31° to 34° north, the drought was excessive from May until late in October. The mercury in Fahrenheit ranged between 85° and 95° in the shade for about three hours after meridian; and at the coldest part of the nights between 75° and 80°. In towns and cities the reflected and radiated heat would increase the extreme temperature to 88° and 96° by day, and 78° to 83° by night. In the latitude of New Orleans, and within 50 miles of the Gulf of Mexico, the seabreeze reduced the temperature two or three degrees, as we ascertained by comparing Dr. Tooley's tables with those kept in the Charity hospital in New Orleans.

After the 10th of August the weather became still more dry and calm: showers ceased to refresh the air, even for a few minutes. The whole region between lat. 30° and 36° was literally parched with drought. Before the last of August the surface of the earth, to the depth of several feet, had become entirely deprived of all ordinary moisture; vegetation began to droop; creeks and small water-courses became entirely dry, or lower than they had been for many years; the cotton plant began to shed its leaves and forms; many forest trees likewise began to shed their leaves. The same condition of weather continued through the months of September and October: the earth was parched; grass, and small vegetation entirely withered and died, under the burning sun; the earth thrown up from the bottom of cistern-pits 14 feet deep, was as dry as dust; wells 60 feet deep, which had never been known to fail, became entirely dry; the cotton plant, in thin uplands, was as completely stript of leaves as if a killing frost had passed over it; the atmosphere continued hazy and smoky for nearly two months; many of the forest trees had shed half their leaves a month before frost; exhaustion and fatigue followed the most moderate exercise in man or beast; the sun beamed down intensely without the intervention of a cloud; large tracts of trees in Opelousas swamps entirely died.

During the months of September and October, the general atmosphere was unusually *calm* and *sultry;* not a breeze moved the stillness of the forest, by day or by night; the morning dews ceased to appear; the stars at night shone with peculiar brilliancy and twinkling. For weeks after the general air was agitated by gentle breezes, the smoke still lingered in the vallies.

This state of the weather existed from the northern limit of Georgia to the western part of Texas, through a zone of six degrees of latitude at least.

The following meteorological synopsis is made from the tables of Dr. Tooley of Natchez:

*June.*—This was one of the hottest months ever known in Natchez: extreme temperature in the shade from 90° to 96° for 21 days; intense unclouded sun; little or no wind, and only three or four light showers;—average temperature at 6 A. M., was 75°; at 12 M., 84°, and at 6 P. M., 88½; lowest point during nights from 70° to 80°.

*July.*—This month presented only a continuation of the same temperature, and general condition of the air.

*August.*—Same general state of weather; innumerable white cumulus clouds; intense sun; one light shower; no wind; average extreme temperature in shade, at 4 P. M., 88° to 90°, and for eight or ten days the mercury ranged from 90° to 94°—nights warm and sultry—not one breeze; last 15 nights between 65° and 70°.

*September.*—Temperature nearly the same, but more calm and sultry; air oppressive for violent exercise, hazy and smoky; three light showers; average extreme temperature by day 85° to 90°; lowest average temperature at night from 70° to 75°; no wind.

The following synopsis is taken from the meterological tables kept in the Charity hospital at New Orleans, where the reflected heat of the city exerts but little influence.

*June.*—Every day is noted as calm; only two light showers; four days are noted as cloudy; for the last 20 days the mercury ranged at noon from 80° to 86°

*July.*—First 20 days were moderately warm—extreme height of mercury ranging from 80° to 88°, at noon; during the last 10 days mercury ranged up between 82° and 88°; 23 days are noted as calm, and 10 of these as *foggy;* four days are noted cloudy with light showers; moderate wind on 6 days.

*August.*—This whole month was *calm*, *dry*, and *foggy;* every day is noted calm; yet at some hour of the day, before the 12th, there was some wind every day, with transient showers; on six days the mercury ranged as high as 90°; near the wharves it would have been 93° or 94°.

*September.*—This month is much as the last—calm, dry, and foggy; every day noted *calm*, 30 days; two light showers; breeze one day.

*October* continued the same, until the 10th, when a strong wind or gale swept along the coast south of lat. 31°.

Such was the general "atmospheric predisposition" to epidemic yellow-fever; and notwithstanding this strong "epidemic constitution," throughout all the south, the whole country, up to the actual introduction of yellow fever cases from the West Indies, was *more healthy than usual* at any season. Not a case of yellow fever was known either in town or country, until it had been prevailing to an alarming extent among the shipping in Charleston, New Orleans, Mobile, Savannah and other ports trading with the West Indies. Not the semblance of disease was seen in any point, until the "*seminarium*" and the "*leaven of infection*" was introduced into a few principal trading maritime ports. Several weeks after these ports became infected, other towns became infected from them, as they had been from the West Indies.

The doctrine which distils a peculiar yellow-fever miasm from marshes, from putrescent animal or vegetable matters, from noisome vapors and noxious odors, exhaled from the surface of the earth, or from any combination of either of these supposed causes, must yield to the facts presented by the whole season of 1839, and by all the epidemics which appeared south of lat. 34°; for in no one instance did the concomitant circumstances accord with the prominent postulates of those theories. The general circumstances of the summer of 1839 are sufficient, we confidently believe, when duly considered by our most eminent medical men, to convince them that the doctrine of the domestic origin of yellow fever in the United States, is entirely unfounded in fact, and utterly confuted by all the facts presented in this epidemic prevalence of yellow fever; the facts themselves, if investigated, demolish the do trine. Some of these we will notice as we progress.

In our examination of the facts, and in the arguments and legitimate deductions which we shall draw from them, we ask of our professional brethren a calm, patient, and impartial consideration. Our object is not to sustain a favorite theory, but to present the facts as they really exist, and to set them forth in their *true light*, so that those medical men, who, by a residence remote from yellow-fever regions, are precluded from personal observation, may have a fair opportunity to examine for themselves, whether the *whole matter has not been misrepresented* to them by most persons who have attempted to treat upon the cause of this pestilence in the United States. We know and readily admit, that the early doctrines instilled by our venerated professors, when we are upon the threshold of the temple of science, adhere to us with all the pertinacity and prejudice with which we would defend the religion of our fathers, right or wrong. Thus with the doctrine of local origin; having by a most unfortunate influence been adopted, it is with great difficulty that any thing having an opposite tendency can have a reluctant examination. We do sincerely believe, that in relation to yellow fever in the United States, the medical faculty have had the subject presented to them in an *erroneous light*; and of no epidemics, more than those of Natchez. In the collection of facts relative to the introduction of the yellow fever into the southern ports and towns, we have derived our information from persons of intelligence and discrimination.

The great point to which all our facts and arguments tend, is to show that *yellow fever is a tropical disease*, peculiar to a few *tropical West India ports;* whence it is occasionally *imported*, in some form or other, into our maritime ports; and that from them it is subsequently *transported to other ports;* that it is *a commercial exotic*, temporarily transplanted upon our soil, *but not indigenous.*

It has been witnessed repeatedly, that during the strongest "epidemic constitution" as it is called, there has been no yellow fever in the United States, unless it has been prevailing to an alarming extent in Havana or some of the West India ports for many days previously. This has been exemplified repeatedly in Charleston, Mobile, and New Orleans. The same principle is confirmed at Natchez, which never has been visited with yellow fever, until *after many cases had been introduced from New Orleans.* Indeed as before remarked, this general hot, dry, and sultry condition of the air in the south is a *certain* and *infallible* indication of a healthy population—exclusive of imported infection.

We desire it to be remembered, that we do *not* advocate the *absolute and unconditional contagion*, or infection of yellow fever, and that it has the property of communicating itself from one individual to another, in a pure and free atmosphere; very far from it. But we do contend, that, under certain circumstances, independently of all local accumulations of city filth, the local atmosphere of towns or cities becomes so contaminated by a healthy population, that it becomes peculiarly adapted to the dissemination and spread of yellow fever, when a portion of infected air is introduced. At some times a moderate quantity introduced will produce this effect; at other times, when the atmosphere is less prepared, a larger quantity is requisite.

We cannot admit, besides the general "epidemic constitution," which *rests equally over town and country*, that a mysterious *tothion* rests over a certain number of doomed points, while all others are exempt from this peculiar influence. In periods of epidemic visitation we find those towns only specially exempt, which by nature are cut off from direct intercourse with infected ports. This principle was exemplified in a most remarkable manner in the summer of 1839, in the lower regions of the Mississippi.

The pure atmosphere of maritime ports, such as New Orleans, Mobile, Charleston, and St. Augustine, is generally such, that yellow fever, when it is introduced, and becomes epidemic, is always far less malignant and fatal, than it is in inland towns, beyond the influence of the sea-breeze. Hence the disease in Natchez, Vicksburg, and Augusta, is more fatal than in the cities on the seaboard. On the same principle, it requires a much greater amount of imported infection, to bring on an epidemic in these cities than in the inland towns; hence the disease will prevail for weeks among the shipping in port, before it spreads among the resident population: but in the interior towns three or four infected steamboats, or a dozen yellow fever cases introduced, within ten days, with their bedding and cloths, will generally produce a sudden out-break of yellow fever as an epidemic. Besides, we always find that the epidemic ceases, in the maritime ports, sooner, and with a less degree of cold, than what is necessary to destroy the infection in inland towns; and in New Orleans and other maritime ports, the proportion of recoveries is much greater than in the inland towns, a large proportion of the cases being comparatively of a milder grade. The atmosphere of *interior towns becomes more contaminated*, and more stagnant, and produces a *more malignant grade of disease*, than the purer air near the seaboard.

In further tracing the origin and spread of yellow fever during the summer of 1839, we will first speak of its appearance and prevalence in New Orleans, and its extension from that city into the lower valley of the Mississippi.

We have already made reference to the fact that yellow fever cases were seen among the shipping in this port, as early as the month of June; and that cases continued to appear among the vessels during the whole of July and until the 12th of August, when it was spreading among the resident population near the wharves. On the 15th of August it was admitted to be epidemic in the city, and strangers were advised by the public authorities to leave the city. The disease spread with great violence over a large portion of the city, near the wharves. Cases multiplied rapidly. A number appeared near the canal basin, among the sailors, draymen, and laborers. Near the river wharves, where the principal shipping lay, the first victims were draymen, clerks, merchants, laborers, and others who were daily in the vicinity, or on board of infected vessels. The Charity hospital became crowded from the first of August until the close of the epidemic. The number of cases admitted into the hospital were as follows: viz., in August, 507; September, 361; October, 110; November, 87.

The report of cases in the Charity hospital is considered a good criterion of the prevalence of the disease, and is published for the information of the citizens. The greater part of the admissions into this institution, consists of seamen, foreigners, and destitute poor, who comprise two thirds of all the cases of fever in the city during the first month of its prevalence.

So soon as these reports corroborate the general impression of alarm, at the spread of the disease, all those who are able and willing, leave the city and seek some retreat among their friends in different towns in Louisiana and Mississippi, within 300 or 400 miles of New Orleans; some retire to the Bays of St. Louis and Biloxi, east of the city; some to the settlements on the Teche, Lafouche, in Oppelousas, and in the towns along the Mississippi as far as Natchez and Vicksburg.

The number of persons of all kinds who leave New Orleans suddenly, or within fifteen or twenty days after yellow fever is announced as epidemic, is seldom less than ten or fifteen thousand, and often not short of twenty thousand souls. Every steamboat is crowded; and every town on the river receives its proportion. Among them are great numbers of Irish and German emigrants, fresh from Europe, many of whom seek employment in the towns above.

As to the *cause* of the epidemic in *New Orleans*, we have already admitted a general "epidemic constitution of the air," such as prevailed over all the southern part of the United States and Texas. But this alone did not produce the disease in this city. Notwithstanding the frequent cases of yellow fever which had appeared among the shipping, as early as the last of June, and the great increase of those cases, during the whole month of July, the *population of the city*, resident and transient, never was more healthy *at any season of the year.* This fact gave assurance of an exemption from an epidemic to those who believe in the local causes to which yellow fever has often been erroneously traced. The public press congratulated the city upon the favorable prospects, and the *absence of local causes of disease.** On the 10th of August a change was admitted by the press, and on the 12th the existence of yellow fever as an epidemic was publicly avowed, and strangers were advised to leave the city.†

By a reference to the port-register of New Orleans it will be seen, that, during the month of July and the first ten days of August, in 1839, *more than twenty-five vessels* arrived in port from Havana, Vera Cruz and other infected West India ports. Most of these were small, such as brigs and schooners, and had left Havana and other ports after the 23d of June, the very time when yellow fever was most malignant in those ports.

We do not intend minutely to trace the introduction of yellow fever into New Orleans, but only to make a few general remarks on the subject of its introduction. It cannot be con-

*N. O. Bulletin, from Aug. 1st to 7th, 1839. †Bulletin, Aug. 12th.

tended by any one, certainly, that filth and such matters gave rise to this epidemic; for during the first week in August, as well as previously, the press was boasting, and justly too, of the unusual cleanliness of the city. Only a few days before the disease spread from the shipping to the resident population, the daily newspapers confidently predicted an exemption from disease, because of the absence of its usual sources, which they erroneously supposed to be putrescent matters about the wharves, and city filth. Another criterion upon which the prediction was based, was the extraordinary state of the health of the city generally.

But a few days showed the fallacy of that theory which attempts to trace epidemic yellow fever to city filth and other local causes. The disease which had been confined chiefly to the shipping, suddenly enlarged the sphere of its action, after a few days of hot, *calm and sultry* weather after the first of August.

We believe that independent of imported infection and cases of yellow fever, New Orleans is naturally as exempt from yellow-fever epidemics as any other port in the United States.

Yet when this city does become strongly infected, it becomes a source from which many other towns and cities on the lower Mississippi are liable to become likewise infected. If in our progress we give any good reasons to convince the reader that yellow fever may be, and has been transported from New Orleans to inland towns, we hope the converse of it may be admitted as true, viz:—that it may possibly have been imported into New Orleans.

We will pass on to the extension of the disease to many towns and villages on the Mississippi, and in the interior of Louisiana, during the summer and autumn of 1839. We think the facts will justify the inference, that in every instance where yellow fever appeared among the population of any of these towns, it was *the result of unrestricted intercourse with New Orleans*, after that city became strongly infected. The intercourse by means of which this disease was introduced into the interior towns, is carried on chiefly through the agency of steamboats, which have been the principal means of extending that disease on the Mississippi above New Orleans.

During the summer and autumn of 1839, many of the towns and villages on the river within 400 miles of New Orleans, and also some towns on other streams in Louisiana, were visited by yellow fever within 20 or 30 days after it became epidemic in the city. In every instance where this occurred, the *first cases* of the disease were invariably traced to New Orleans; and *only such towns* as had free intercourse with the city by steamboat, were visited by the disease. Those towns which were cut off from such intercourse by nature or circumstances, invariably escaped the epidemic, although they might have double the population of others, and might be only half the direct distance from the city. The first individual cases, in any of these towns, were either persons landed from steamboats with the disease openly developed in their systems, or persons who had recently left New Orleans with the infection dormant in their systems when they landed, but which soon after was developed in its most malignant form.

The order in which the interior towns became infected, was that of the extent of their commerce and the frequency of steamboat intercourse with the city of New Orleans. Not a single town or village presented a case of yellow fever until 15 or 25 days after it had been fearfully epidemic in New Orleans. Yet all these inland towns had been exposed to the same "epidemic constitution" of the atmosphere, and even in a much higher degree, as we have already shown in our remarks on the weather. If some foreign agent or influence were not necessary, why did the epidemic visit New Orleans one month earlier than Natchez or Vicksburg?

Again we repeat that in no instance did a case of yellow fever occur, as a harbinger of an epidemic, in any of the interior towns, unless such case was clearly traceable to New Orleans infection. In every instance too, the disease began to spread first about the wharves and steamboat landings. Examine a map of Louisiana and Mississippi closely, and no one town can be pointed out which was entirely cut off from communication with New Orleans, and which had any cases of yellow fever; even towns on tributary streams, which carry on a large commerce with the city in spring seasons, but which were cut off by low water in August and September, were as free from yellow fever as those which never had any such intercourse. On the other hand, *every town and village* on the Mississippi, and in the interior, within 300 or 400 miles, which had unrestricted commercial intercourse with New Orleans after the 20th of August, suffered more or less from epidemic yellow fever

As we have made frequent allusions to the agency of *steamboats* in spreading the disease on the lower Mississippi, it may be proper here to explain more particularly the manner in which this effect is produced.

The introduction of steamboats upon the Mississippi, has produced a new era in the commerce of this great valley. Formerly vessels from Havana and other ports, were often 10 and even 20 days in ascending the river to New Orleans, propelled by wind alone, against the strong current and the numerous bends of the river. Now they are towed from the Balize in less than 24 hours. But the greatest change has been effected in the commerce on the river *above* New Orleans.

Formerly the whole commerce down the river, was in flat-boats, or floating arks; but few boats, comparatively, *ascended* the river, and these were barges and keel-boats, propelled by hand, at the tardy rate of eight or ten miles a day, and often less. A barge would be 30 days from New Orleans to Natchez; and one barge did not leave the city for the towns above, where twenty steamboats do now. Of course the ascending commerce of the city is a hundred fold more than it was before the introduction of steamboats. Scarcely a day elapses without the arrival of from one to five boats, ascending direct from New Orleans; and they continue their trips during the prevalence of the epidemic. The packets in the lower trade continue their trips regularly all the year, and make regular landings at all the important landing-points above New Orleans.

These boats, after lying at the wharves for several days each trip, in the midst of the infected air of New Orleans, when the epidemic is prevailing, and after receiving their freight from the infected district, besides probably half a dozen cases of yellow fever in the meantime, at length become badly infected, and the entire atmosphere, in confined portions of the boat, becomes as thoroughly infected as the air of the infected district itself. A boat may thus contain an infected atmosphere, which may infect one or more persons at each village or landing where she touches, provided they go on board and remain a few minutes. In this manner persons visiting a boat may contract yellow fever with as much certainty as if they had visited the infected district of a city for an equal length of time. A steamboat is a large moving tavern, and may become as much infected as any stationary tavern or hotel in a city; and those boats which remain at the wharves of New Orleans for five or six days, discharging and receiving freight from the infected district, after the epidemic assumes its most malignant character, are fortunate if they do not become infected by the first or middle of September. At every town and village where the boats make a landing, there are scores of persons of all descriptions, who immediately rush on board where they remain until the boat is about to leave. If one, out of every ten, contracts the disease, the continual arrivals will soon infect twenty or thirty persons, who will probably sicken at different periods, from three to eight days afterwards. These may reside in opposite parts of the town, and each may be traced erroneously to some contiguous collection of animal or vegetable matter. Twenty or twenty-five cases thus contracted, in a strongly contaminated atmosphere, may be sufficient to produce a sudden outbreak of epidemic yellow fever.

Without multiplying examples, we will adduce the case of the steamboat Corsair, which left New Orleans in September 1839, for St. Louis, having on board at least 50 passengers of all kinds, besides her crew. Soon after her departure many of them began to sicken with yellow fever daily, and one or two died every day during her trip. Before she arrived at St. Louis, this boat had buried fifteen of her passengers and crew at different points along the river bank. Others recovered, and many were still sick when she arrived at St. Louis. Most of these persons, no doubt, contracted the disease in New Orleans; but such a number of cases was sufficient to infect the boat, especially after receiving her freight from the infected district. Had this boat continued to run in the lower trade, in all probability, before November, she would have been instrumental in spreading yellow fever in many towns on the lower Mississippi. This is not an extreme case by any means in this region; and it is beyond doubt true, that *yellow fever was unknown in the towns above* New Orleans previous to the introduction of steamboats.

After New Orleans had become strongly infected, and the epidemic had been prevailing fatally for about two weeks, the disease began to make its first appearance at various points on the river above; and even on Red River, and the bayous south and east of Oppelousas. The points where it first appeared in this manner, were the towns and landing-places most intimately connected with New Orleans by steamboat navigation.

The following are the principal points on the Mississippi river above New Orleans; viz.

| | |
|---|---|
| 1. Donaldsonville, | 5. Bayou-Sara, |
| 2. Plaquemenes, | 6. Fort-Adams, |
| 3. Port-Hudson, | 7. Natchez, |
| 4. Waterloo or Pointe Coupeé, | 8. Vicksburg. |

Besides those places immediately upon the banks of the Mississippi river, there were the following towns and villages on Red River and the bayous of lower Louisiana; viz.,

| | |
|---|---|
| 1. Alexandria, | 5. New Iberia, |
| 2. Natchitoches, | 6. Franklin, |
| 3. Oppelousas, | 7. St. Martinsville, |
| 4 Thibedeauxville. | |

The disease as it appeared in each of these towns, was clearly traced to New Orleans, for the first cases, during a week or ten days, and until it became epidemic, after having established a new centre of infection; which then produced an extension of the disease independent of any *additional importation.*

1. *Donaldsonville.* This town is situated on firm alluvial banks, on the west side of the Mississippi, and immediately below the efflux of the Lafourche, 85 miles above New Orleans. It is a beautiful and cleanly town, with a population of about 1000 souls. In high stages of the river, steamboats run regularly from New Orleans into the Lafourche, and to the whole settlement on that stream; but in low water, the outlet being dry, all freight and passengers for the Lafourche are landed at Donaldsonville; whence there is a short portage to the deep water in the bayou a mile or two below, where other boats receive them.

During the summer of 1839, Donaldsonville, like all other towns on the lower Mississippi, was remarkably healthy until the first of September, when yellow fever had been epidemic in New Orleans for more than two weeks. This state of health continued *uninterrupted*, until after ten or twelve cases of yellow fever had been introduced from New Orleans by the boats; besides a few persons who arrived from the city with the infection dormant in their systems, and soon after were attacked by fully developed yellow fever. These cases were all taken to the public hotel or to other houses in that vicinity, and near the steamboat landing. At length towards the middle of September, the local atmosphere was contaminated, or *infected;* and other persons, who had not been exposed to any other source of infection, contracted yellow fever and died, after having been more or less in the newly infected district. The remainder of the town continued healthy. Among the first persons attacked in Donaldsonville, after the first imported cases, were several persons who had visited, nursed, and sat up with the sick. The disease continued to spread slowly until frost, when about 30 deaths had occurred besides the first imported cases. This statement is given upon the authority of Col. H. T. Williams, Surveyor General of Louisiana, and of Mrs. C. M. Thayer, both residents of that town. It will be remembered that Donaldsonville, during that period, was a regular depot for the trade of all the Lafourche country; and that passengers and freight were obliged to remain there from one to three days before reshipment. Not being a commercial town, it is destitute of wharves and other supposed sources of yellow-fever miasm.

2. *Plaquemines.* This is a small straggling village along the west bank of the river, just below the outlet of the Plaquemines bayou, 34 miles above the last. It contains a population of 25 or 30 families, besides a few stores and warehouses for the Plaquemine and Oppelousas trade. At low stages of the river it becomes a depot and carrying point for the trade; the outlet of the Plaquemines is dry at such times, and steamboats cannot pass through.

This village was entirely free from any disease until after several yellow fever patients had been landed by the boats from New Orleans; besides some few persons who sickened and died on their way to Oppelousas. A local air became infected; and the disease was thereby communicated to others who had not been exposed to any other source of infection. Twelve or fifteen persons died at this place of yellow fever.

We should have remarked before, that when any town becomes infected, or when a few cases of yellow fever make their appearance, the *mass of the inhabitants immediately desert the place;* and the cases which occur, as well as the deaths afterwards, are from among the remnant of population who decline leaving.

The next town on the river above is *Baton-Rouge,* on the east bank, upon high rolling ground, 140 miles above New Orleans, and 23 miles above Plaquemines. The population of this town is about 8 or 900. This is *not a trading town,* and no extensive settlements are near to make it a shipping point. The United States' Arsenal, State Penitentiary, and other public buildings, give it all its importance. This town in 1839, like some others which we shall hereafter notice, *entirely escaped the epidemic yellow fever*, by a *quasi non*-intercourse with New Orleans, during the epidemic in that city. Besides the absence of a trading population, there is a shoal bar near the town, which prevents steamboats from landing in low-water; and further, the people, unbiassed by the interested policy of a *mercantile* community, and left to the deliberate convictions of their own judgments, refused to receive yellow-fever patients into their town. Their apprehensions had been thoroughly awakened to the danger of *imported* yellow fever, by an occurrence which was still fresh in the memories of many of them. I allude to the fact, that in the fatal autumn of 1829, a number of Spaniards, refugees from Mexico, had sought refuge from political dangers in New Orleans; but soon after their arrival, the yellow fever made its appearance in that city, and they were obliged to retire to a place of safety from disease. They took a steamboat for Baton-Rouge, after many of them had contracted the seeds of disease in the infected city. Soon after their arrival at Baton-Rouge, yellow fever made its appearance among them, and many of them perished. An infected atmosphere was generated, which communicated the disease to the resident population, among whom it prevailed with great mortality. For these facts I am indebted to D. P. Cain, Esq., of East Baton-Rouge parish.

This *non-intercourse*, and the *non-introduction* of yellow-fever patients into Baton-Rouge in 1839, is the only satisfactory reason *why this place escaped* during this fatal epidemic season; when every town and landing place below, and above, for 250 miles, with *uninterrupted intercourse*, were desolated by the pestilence.

3. *Port-Hudson.* This is a small village containing about 30 houses and 100 souls. It is situated on the east bank of the Mississippi, upon a firm clay bluff, about 35 feet above the river alluvion, and 25 miles above Baton-Rouge. It is the shipping point for an extensive back settlement, 25 or 30 miles distant. A rail-road from Jackson, La., intersects the river at this point, which also contributes to render it an important steamboat landing. During the month of September, the yellow fever was introduced among the merchants, clerks, and laborers, and about fifteen of them died. Others from the country contracted the disease.

4. *Waterloo.* This is an important landing-place for the rich settlements on Fausse Riviere, of Pointe Coupeé. It is five miles above Port-Hudson, and is situated on the west bank of the river. The commercial intercourse between this place and New Orleans was *uninterrupted;* and besides the usual steamboat communication, during the epidemic in the city, a number of the French inhabitants, believing they possessed a constitutional immunity against the disease, made a visit to New Orleans in the midst of the epidemic. After a few days of pleasure and dissipation in the city, they returned; and several of these were soon attacked with yellow fever and died. An infected atmosphere was generated, and several others, who were not exposed to any *other* source of infection, sickened and died. The whole number of deaths at this place was about 15. I derive this information from D. P. Cain, Esq.

5. *Bayou-Sara.* This is a very important shipping point, on the east bank of the Mississippi, about 6 miles above the last village. The East Feliciana rail-road terminates at this point. The principal town, St. Francisville, is nearly a mile from the river, on high rolling ground; while Bayou Sara is situated on the immediate bank of the river, and is properly the landing, or shipping point for the town, and an extensive settlement for 50 miles back.

When yellow fever became epidemic in New Orleans, many persons came to Bayou Sara, and the vicinity, as a retreat from disease; others arrived at intervals subsequently; and the regular packets, besides the boats in the upper trade, continued their trips as usual during the epidemic, until many cases of yellow fever were introduced, as at other points. An infected district was produced near the steamboat landing, and the disease finally spread among the resident population; it was also communicated to some from the country, who had not been exposed to any other source of infection. About 20 persons died in this town and its vicinity. This place, as well as St. Francisville, was remarkably healthy until after cases of yellow fever were introduced from New Orleans.

6. *Fort-Adams.* This town is situated on the immediate bank of the river, on the east side, at the foot of a high hill or bluff, many of the houses being crowded up the side of the hill. It is 75 miles above Bayou-Sara; it is a town of considerable trade for the back country, and is a shipping point for steamboats. The population is about 300. The yellow fever was introduced into this town in the same way that it was introduced into Bayou Sara and other towns below. It assumed an epidemic form late in September, and about 20 deaths occurred before it was checked by frost.

7. *Natchez* is the next in order. This city is nearly 300 miles above New Orleans, on the east side of the river. It is situated chiefly upon high ground, nearly 200 feet above the river; a portion at the immediate landing for steamboats and flatboats, is at the base of the bluff, along a narrow strip of alluvium, about 50 yards wide, and 600 yards in length. This portion of the city contains about 50 or 60 houses, including several large warehouses, stores, and hotels, with a resident population of nearly 200. It has always been the point first infected with yellow-fever, with one exception, which is easily explained. The disease always makes its first appearance among the clerks, shop-keepers, laborers and others, who reside upon the wharves or frequent the steamboats during the summer season.

Before we proceed to speak further of the epidemic of 1839 in Natchez, we will take occasion here to remark, that the beautiful *village of Vidalia half a mile distant on the opposite side of the river, has never been known to be visited by yellow fever*, even when it has been raging fearfully in Natchez. It has always continued healthy although Natchez, on the opposite bank, was perfectly desolated by that disease. The situation, as regards local causes, is in no wise different from

Donaldsonville, Plaquemines, and Waterloo, except that Vidalia *is not* a trading town; steamboats are scarcely ever known to make a landing there. Natchez, immediately opposite, is the port; and Vidalia has no more intercourse direct with New Orleans by steamboat, than if it were ten miles from the river. Hence, we infer that *Vidalia owes its exemption solely to non-intercourse* with New Orleans by steamboats. Even in 1839, when the "epidemic constitution" of the atmosphere was universal in this latitude, Vidalia was perfectly healthy. Why this peculiar exemption? The only valid reason to be assigned is, that the *indispensable requisite* is absent —the *leaven of imported infection*, which is introduced by steamboats. Vidalia is in the midst of an extensive alluvion, and as much exposed to marsh miasm as New Orleans itself, and far more than Natchez.

In regard to the state of the weather at Natchez in 1839, we have already shown that it was excessively *warm*, *dry*, and *sultry*, with a smoky or hazy atmosphere. During the last 15 days of August, the mercury ranged as high as 90° and 91° each day, in the shade; and there was but little change from that during the whole month of September. Even the month of October was characterized by continued hot, sultry weather, with the mercury ranging almost as high.

*Natchez never was more healthy* than during the last 30 days preceding the epidemic of 1839. Without fear of contradiction, I might say there was no city in the United States, of the same population, more healthy than Natchez was, until after the introduction of yellow-fever cases, and constant steamboat intercourse with New Orleans for nearly 30 days after yellow fever had been epidemic in that city. The whole country was alike healthy; *not a case of bilious fever was known;* even the negroes who toiled in the sun, on the hills of Mississippi, and in the swamps of Louisiana, were alike free from the usual diseases of the season. At such a time as this, our citizens were daily exposed to imported infection, for nearly 30 days after yellow fever had been epidemic in New Orleans. During that time, scarcely a day elapsed, without the arrival of one or more steamboats crowded with people returning to the north, besides scores of Irish and German emigrants direct from New Orleans with their bundles of filthy clothes and beds, and occasionally one or two open cases of yellow fever to be sent ashore to the hospital. More or less of these foreign emigrants were left by each ascending boat, and often with the seeds of disease dormant in their systems and ready to be developed in a few days in the midst of a healthy population; steaming infection from the close and crowded huts and cellars in which they were compelled to seek shelter. Before the 20th of September there had been twenty deaths in the hospital, chiefly from among this class, and from the sick taken off the boats. Besides these, there were several persons residing in Natchez who had imprudently visited New Orleans, and who were attacked soon after their return.

Such was the state of things in Natchez for more than three weeks before the epidemic broke forth in its fury. Under these circumstances, and with the daily importation of large quantities of blankets and woollen goods for the planters, all from the infected district of New Orleans, could any one reasonably expect *less than an epidemic yellow fever*? Southern people, who are unbiassed by interest, know too well the character and habits of this disease to plead ignorance on the subject. If, knowingly, they permit their better judgment to be influenced by a class of interested merchants, and suffer pestilence to be imported and spread among the helpless and innocent population, the innocent and the guilty must suffer together. But the municipal authorities must answer to God and their fellow citizens for the untimely deaths of hundreds of helpless poor, and many valuable citizens.

After many cases of yellow fever had been introduced and others had occurred, there seemed suddenly to be two principal centres of infection in the upper part of the city. These were the City Hotel, and the Railroad Hotel, both within fifty yards of the west end of Main street. The former was the resort of American strangers generally; the latter, in like manner, was the resort of the German emigrants, of whom there were many in the city. Each of these hotels were more or less crowded with strangers, daily arriving from New Orleans.

Yet in these houses and vicinity, an infected air was not produced until late in September, after the disease had prevailed near the steamboat landing for nearly two weeks, and several persons had died at the hotels.

Here we would remind the reader that in 1837, the first principal focus or centre of infection in the upper city was a German tavern on the corner of State and Commerce streets, which at that time was the principal resort of the German strangers. This custom was transferred to the Railroad Hotel in 1839, when the former was discontinued. In 1839 that portion of the city near the corner of State and Commerce streets was almost exempt from the disease; while in 1837, there being no such house as the Railroad Hotel, that point was comparatively exempt from the disease.

It was not until the 22d of September, 1839, that the disease began to spread rapidly near the steamboat landing; immediately after which a large portion of the population fled to the surrounding country for protection, and by the 28th the population of the city was reduced to 800 or 900 souls. The epidemic raged with great malignity until the middle of November, when 235 persons had died, viz:—69 in September, 135 in October, and 31 in November. Three practising physicians died, and three recovered after the most severe attacks. The whole number of cases was about 500.

The advocates of its local origin from city filth and putrescent matters, or from decaying vegetables, or miasm, were compelled to abandon that ground as *untenable* in the present epidemic. The facts relative to the beginning and spread of this epidemic, made hundreds of proselytes to the doctrine of imported infection, which could never have been effected by human reasoning.

Such had been the reliance upon a cleanly condition of the city, as an infallible guarantee of health, and as security against an epidemic, that the municipal authorities, in accordance with these views, had caused the whole city to be thoroughly cleansed and spread with lime as a preventive. So effectually had this been accomplished, that the advocates of the local origin confidently predicted an exemption from an epidemic visitation, and derided the very mention of the word *quarantine.* The malignant epidemic which soon after broke out, convinced them that *something besides city filth, and the like, could* produce epidemic yellow fever.

It may be inquired, why is Natchez so frequently visited by yellow fever, when Vicksburg and other towns above Natchez have frequently been exempt? The reason is clear and unequivocal to my mind. Natchez is about 300 miles above New Orleans, a run of 36 or 40 hours for ordinary boats. It is the first port of entry, and by far the most important commercial point between New Orleans and St. Louis. It is the only point in this whole distance where a public hospital is prepared for the reception of sick and indigent boatmen, and yellow-fever patients from the ascending boats. Scarcely a boat from New Orleans passes Natchez without making a landing, at all seasons, and especially during an incipient epidemic in New Orleans, to discharge the numerous passengers and emigrants flying from the disease, as well as to relieve themselves of the sick who may be on board, either among the cabin or deck passengers. Hence every boat ascending to St. Louis, or the Ohio, is sure to make the *first principal landing at Natchez,* and the packets frequently make it the termination of their upward trips. Every boat making such a landing at Natchez, has no occasion to stop again at Vicksburg, or the intermediate towns; because her principal passengers, not bound for the upper country, *as well as all her sick,* are discharged at Natchez. Before other cases of disease are developed, the boat is in a colder region, or far above Vicksburg. In such cases, it is common for the boats to stand out in the river, at Vicksburg, for a few minutes, until the yawl only is sent ashore.

Hence, when the hospital at Natchez, (*which is within the city*) is kept open for the reception of yellow fever patients, it *serves as a lure* to attract infected boats to her wharves, and *invite pestilence among her own citizens.* This very policy often protects the towns above and below, while it exposes her own citizens to the horrors of a malignant epidemic.

The converse of this has been clearly proven in two epidemics—those of 1839 and 1841. In 1839, Vicksburg, the next important town above Natchez, continued free from yellow fever until after Natchez had become so thoroughly infected and desolate, by the 10th of October, that the upward-bound boats ceased to make landings there, and passed on to Vicksburg, 110 miles above, as their first principal landing above New Orleans. What was the result? *In fifteen days from that time, yellow fever was epidemic at Vicksburg,* in all the lower part of the city near the steamboat landing. Again, in 1841 Natchez closed her hospital and established a quarantine as soon as yellow fever was epidemic in New Orleans. What was the consequence? The first principal landing for upward-bound boats was at Vicksburg, which continued healthy up to that time, but was visited *with a most malignant epidemic* within twenty days after the Natchez quarantine was enforced; while Natchez, thus protected, continued perfectly healthy during the whole time the epidemic was raging on both sides of her.

Here I will cite another argument somewhat analogous, in relation to the protection of *Washington,* six miles east of Natchez. We have already, in the early part of these observations, spoken of the malignant epidemic which was introduced into Washington from Natchez in 1825. In 1839 all parties agree fully in the "epidemic constitution" of the general atmosphere on the lower Mississippi; this existed in Washington, no less than in Natchez. Such was the state of things when the people of Natchez fled at the first alarm of yellow fever in 1839. Washington was one of the principal retreats; and the town authorities, having the scenes of 1825 full in their minds, had watched with anxious solicitude the reckless policy of Natchez, and the gradual introduction of yellow fever into the city, and determined to protect their own citizens. On the 15th of September, when the disease was making its gradual advances in Natchez near the wharves, the selectmen of Washington passed an ordinance *prohibiting the introduction* of all feather-beds, bedding, blankets, and other porous articles, from Natchez, after the 18th of September, believing that the disease would then be epidemic in that city. Yellow-fever patients were expressly prohibited from entering the town. The ordinance was rigidly enforced, and the whole town, with its crowded population, was entirely protected from the threatened pestilence, although about eight cases of disease developed itself in those who came out from Natchez apparently healthy. Every one in the slightest degree acquainted with the habits and approaches of this epidemic, were fully satisfied that it only required the *spark* to ignite the pestilential influence. At that time Dr. Samuel Hogg, formerly of Nashville, expressed to me his extreme fear that the crowded state of Washington would lead to an outbreak of yellow fever. But the whole population continued as healthy as usual, protected, as all admitted, by the "quarantine" as it was called. Those who had witnessed the epidemic of 1825, concurred in the opinion, that the general atmosphere in 1839 was better adapted to spread an epidemic than in 1825. In 1825 the intercourse with Natchez was *unrestricted;* daily supplies of goods, woollens and negro blankets, were received by the merchants. A malignant epidemic followed in 1825, and in 1839 all continued healthy.

8. *Vicksburg.* This is a port of entry on the east side of the Mississippi, about 400 miles above New Orleans. It is situated upon the side of a steep hill, or bluff upland, facing the river, and upon a strip of alluvion at the base of the bluff, about 50 yards wide, and half a mile in length. The latter is crowded with stores, warehouses, produce-stores, and numerous small shops, up to the immediate bank of the river, near the steamboat landing. The population is about 4000 souls.

This city, during the most fatal epidemics which have desolated Natchez, *has always been exempt until* 1839, when only a partial epidemic, which was checked by frost, had commenced. The importance of Vicksburg, as a port and shipping point for steamboats, has been increasing for the last few years; and in 1839 the railroad leading to Jackson was put into operation. This at the time drew a great many Irish laborers to the place when the yellow fever drove them from Natchez; and since the completion of the railroad, the commercial as well as the travelling intercourse has been augmented.

In 1839 this city continued as healthy as any city in the south until about the 10th of October. At that time the lower part of the city, at the foot of the bluff, began to be very sickly, and several deaths occurred every day, until the last of the month, when there had been as many as seven deaths per day for a part of the time. None could deny that some malignant disease was daily sweeping off its victims; but as none of the medical faculty of Vicksburg, although equal as a body to any in the Union, had ever seen yellow fever, and did not apprehend any such thing, from the cleanly state of the city, they concurred generally in calling it *congestive fever*. It continued its ravages until checked by frost early in November. By that time, at least 50 persons of all kinds had fallen victims to it; of these deaths about 30 occurred in the vicinity of the steamboat landing and many others near the railroad depot, among the Irish shanties. In the latter place an infected atmosphere was generated, which communicated the disease to those who had not been otherwise exposed.

This disease was, most *unquestionably, yellow fever.* Its symptoms, general character, and issue, have been detailed to me, and places it, in my mind, beyond a doubt. Dr. Hicks, who practised in the midst of it, towards the last of October, assured me that it was genuine yellow fever, with genuine *black vomit.* This peculiar characteristic of the disease cannot be mistaken by any physician who has once seen and examined it. Since the close of the epidemic of 1841 in this city, I have conversed with several of the physicians of Vicksburg, who had become familiar with *yellow* fever, and they were free to admit, that the fever of 1839 was genuine yellow fever. Many cases of yellow fever in the first stages assume such an insidious aspect, that our most discriminating *teachers of medicine,* who have not been familiar with it, would be greatly deceived in their diagnosis, and mortified in their prognosis. Even in the advanced stage, there are cases where one not familiar with it, would pronounce a patient *convalescent* when he was *moribund.*

The first cases of this disease at Vicksburg in 1839, were brought there by steamboats; some were cases openly developed, and others were persons with the seeds of disease in their systems, but apparently in perfect health. An infected atmosphere was created near the steamboat landing, and near the railroad depot, which was gradually extending itself when it was neutralised by frost.

It is a material circumstance in this epidemic, that the city was *unusually healthy* for nearly three weeks after the epidemic had been raging in Natchez, and the first cases were certainly imported. And, had they been imported early in September, the epidemic would have matured before frost, and would have swept off five times the number it did.

How was it in 1841? Natchez established a quarantine; all the boats, with their refugee emigrants, and *yellow-fever patients,* or those with the seeds of disease dormant in their systems, were carried on to Vicksburg, and soon they spread yellow fever *there,* as they *would have done,* and as they *had often done before,* in Natchez. In twenty days, or by the 20th of September, they succeeded in producing epidemic yellow fever in Vicksburg, and it raged with great malignity until the first of November. Hence the first regular epidemic in Vicksburg occurred while Natchez, protected by a quarantine, escaped entirely.

The same principle was exemplified and illustrated, in the case of other towns, and especially Grand Gulf. This town in 1839 permitted free intercourse with all ascending boats which chose to land, and freely admitted the sick, and others. The consequence was that there were about 20 deaths from yellow fever, and the disease was assuming an epidemic form, by generating an infected atmosphere, just as it was arrested by frost. The appearance of these cases in Grand Gulf was simultaneous with those in Vicksburg, and occurred only after boats ceased to land at Natchez. But in 1841, the case was altered. The people of Grand Gulf, encouraged by the example of Natchez, prohibited intercourse with infected boats, and utterly refused to permit yellow-fever patients to be set ashore within the town. Several of the regular packets desired to send some of their crews or engineers ashore for medical aid; but it was resisted by the people. Only two cases of yellow fever occurred in Grand Gulf in 1841, and they were in persons who had arrived from New Orleans, apparently in perfect health, but with the infection dormant in their systems. Grand Gulf continued as healthy as any town on the river.

Our attention will be next called to a brief notice of the disease as it prevailed in the towns on Red River, and upon the bayous west of the Mississippi and south of Red River; and also, incidentally, to its appearance along the northern shore of the Gulf of Mexico, and in Charleston, S. C., and Augusta, Georgia.

(TO BE CONTINUED.)

